# Antibiotic Resistance of *Salmonella enterica* Serovar Typhi in Kolkata, India, and *In Vitro* Experiments on Effect of Combined Chemotherapy

**DOI:** 10.1100/2012/454059

**Published:** 2012-01-29

**Authors:** Shyamapada Mandal, Manisha DebMandal, Nishith Kumar Pal

**Affiliations:** ^1^Bacteriology and Serology Unit, Department of Microbiology, Calcutta School of Tropical Medicine, C. R. Avenue, Kolkata 700073, India; ^2^Department of Zoology, Gour Banga University, NH-34, Mokdumpur, Malda 732103, India; ^3^Department of Physiology and Biophysics, KPC Medical College and Hospital, Jadavpur, Kolkata 700032, India

## Abstract

This communication states the changing patterns of *Salmonella enterica* serovar Typhi (*S.* Typhi) isolates causing enteric fever in and around Kolkata, India. Among the isolates resistance to ampicillin (A), chloramphenicol (C), cotrimoxazole (Co) and tetracycline (T) were plasmid mediated; the plasmid was unstable in *S.* Typhi, and the other enteric bacteria like *Escherichia coli*, Klebsiella pneumoniae and Proteus vulgaris were found to be the potential source of dissemination of such plasmids into *S.* Typhi. The infection with such *S.* Typhi strains were successfully treated with ciprofloxacin (Cp: MICs 0.0075–0.075 **μ**g mL^−1^) and/or ofloxacin (Ofx: MICs 0.0125–0.075 **μ**g mL^−1^), but in the later course, the *S.* Typhi strains, showing resistance to nalidixic acid, developed low level of resistance to Cp and Ofx, causing the treatment failure. Thus, the treatment regimen was shifted to the third generation cephalosporins like ceftriaxone (Ct) and cefotaxime (Cf). Keeping in mind the anticipation of development of resistance to Ct/Cf, we prepared the treatment regimen for MDR enteric fever, based on the double-drug synergy tests in vitro; Cp-gentamycin (FICI 0.121–0.216) and Cp-trimethoprim (FICI 0.14–0.483) combinations were found effective against *S.* Typhi isolates having decreased sensitivity to cp (MICs: 0.5–1.25 **μ**g mL^−1^).

## 1. Introduction

Typhoid fever, the causative agent of which is *Salmonella enterica* serovar Typhi (*S*. Typhi), remains a serious public health problem in many developing countries, with highest incidence in parts of Africa (50 per 100.000 person-years) and Asia (274 per 100.000 person-years) [1, 2]. The period 1990 to the present has been a hallmark era in the history of enteric fever because of the emergence and dissemination of *S*. Typhi strains carrying resistance to multiple clinically relevant antibiotics [3, 4]. In India enteric fever is a major public health problem accounting for more than 300,000 cases per year and *S*. Typhi is the commonest etiological agent. Most developing countries have experienced outbreaks of multidrug-resistant (MDR) enteric fever due to *S*. Typhi; Kolkata (Calcutta) and its suburbs also faced epidemic of enteric fever caused by life threatening infection of *S*. Typhi [5].

The above background prompted us to (i) determine the antibiotic resistance pattern of outbreak causing as well as sporadic isolates of *S*. Typhi (1991–2001) obtained at the Calcutta School of Tropical Medicine from blood cultures from enteric fever patients within and outside Kolkata, India, (ii) determine the dissemination of R-factor of *S*. Typhi isolates, (iii) correlate the zone diameter of inhibition (ZDI) and minimum inhibitory concentration (MIC) values for evaluation of resistance to ciprofloxacin (Cp) and to evaluate the Nalidixic acid (Nx) resistance as a marker for decreased Cp susceptibility in *S*. Typhi isolates, and (iv) evaluate the *in vitro* efficacy of various combination of antibiotics against *S*. Typhi in order to combat antibiotic resistance.

## 2. The Study

The *S.* Typhi isolates showed flip-flop patterns of resistance to ampicillin (A), chloramphenicol (C), co-trimoxazole (Co), and tetracycline (T) with wide range of MICs, 0.1–6000, 0.1–5000, 0.5–1200, and 0.5–800 *μ*g mL^−1^, respectively. A total of 421 *S. *Typhi isolates from blood samples of suspected enteric fever patients undergoing treatment at the Calcutta School of Tropical Medicine, Kolkata (India), were used in the present study; a single strain was isolated from a each of the cases. The control strain used in the study was *Escherichia coli *ATCC 25922.

The presence of R-factor in *S*. Typhi (resistance pattern ACCoT or ACCoTNx) was established by (a) transfer of resistance from MDR *S*. Typhi to the *Escherichia coli *C600 recipients (Nx^R^, F^−^) by *in vitro* conjugation experiments, (b) secondary transfer of R-factor to the antibiotic-sensitive *S*. Typhi isolates, (c) elimination of antibiotic resistance from donors as well as transconjugants by curing experiments, and (d) isolation of plasmid DNAs from the donors, recipients, transconjugants, and cured strains. A conjugative plasmid of approximately 55 kb was associated in mediating ACCoT resistance among the MDR *S. *Typhi isolates, while the antibiotic sensitive isolates were plasmidless.

On the other hand, the antibiotic-sensitive *S.* Typhi strains acquired ACCoT resistance along with the acquisition of a plasmid (comigrated with the MDR *S.* Typhi plasmid) from clinical isolates of MDR *Escherichia coli *(resistance pattern ACCoTNx or ACCoTNxCp), *Klebsiella pneumoniae* (resistance pattern ACCoT), and *Proteus vulgaris *(resistance pattern ACCoT). Resistance to Nx and Cp was not transferable. After curing experiments, the loss of ACCoT resistance from the original MDR *S.* Typhi strains, all types of transconjugant *S*. Typhi strains and MDR *E*.* coli *strains were concomitant with the loss of the plasmid.

The MDR enteric fever cases were treated successfully with Cp (500 mg) or Ofx (200 mg) twice daily for 5 days. The associated *S.* Typhi isolates showed MICs 0.0075–0.075 *μ*g mL^−1^ for Cp and 0.0125–0.075 *μ*g mL^−1^ for ofloxacin (Ofx). The *S*. Typhi isolates (1995 onwards) showing Cp MICs 0.1–1.25 *μ*g mL^−1^ and equivalent ZDI of ≥21 mm caused treatment failure during Cp therapy. We encountered problem in treating enteric fever with Ofx (200 mg twice daily for 5 days) too during and after 1996, although the isolates fall within the sensitive range by disc diffusion method (≥16 mm). The corresponding isolates showed high MICs of Ofx (0.5–1.5 *μ*g mL^−1^). In order to determine the MICs of antibiotics and ZDIs around the antibiotic discs, the methods of the Clinical and Laboratory Standards Institute (CLSI, formerly called the National Committee for Clinical Laboratory Standards; NCCLS) were followed [6, 7].

**Figure 1:**
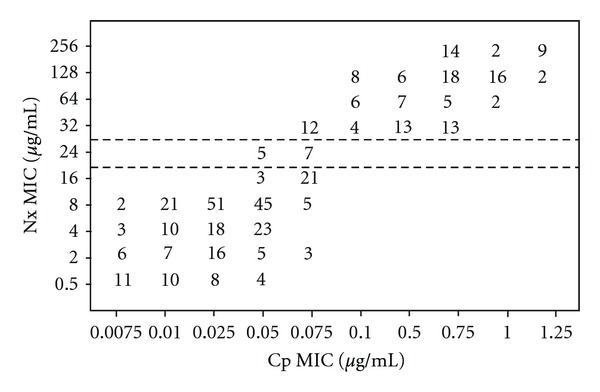
Scattergram for 421 *S. *Typhi isolates correlating the minimum inhibitory concentration (MIC) values of nalidixic acid (Nx) to the MICs of ciprofloxacin (Cp). The broken horizontal lines represent the NCCLS breakpoint recommendations for susceptibility (MIC, ≤16 *μ*g/mL) and resistance (MIC, ≥32 *μ*g/mL) to Nx. The numbers within the graphic indicate the number of *S.* Typhi isolates.

**Figure 2:**
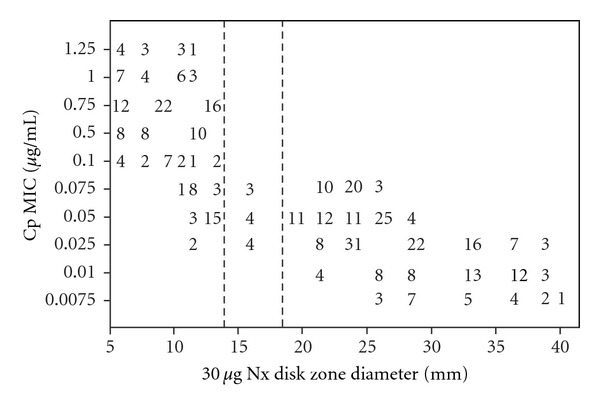
Scattergram plotting the minimum inhibitory concentration (MIC) values of ciprofloxacin (Cp) and zone diameter of inhibition (ZDI) around 30 *μ*g nalidixic acid (Nx) disc for 421 *S. *Typhi isolates processed by the NCCLS methods. The broken vertical lines represent interpretative ZDI suggested for Enterobacteriaceae (susceptible at ≥19 mm, resistant at ≤13 mm). The numbers within the graphic indicate the number of *S. *Typhi isolates.

The relevance of using the resistance to Nx as a marker for Cp resistance in *S.* Typhi isolates was evaluated by comparing the MICs of Cp and that of Nx (Figure 1), and MICs of Cp and ZDI obtained around 30 *μ*g Nx disc (Figure 2). When Cp MIC of ≥0.1 *μ*g mL^−1^ was used as a breakpoint of decreased Cp susceptibility (signifying resistance), the sensitivity of Nx disc was 100%, and the specificity was 89.20%, and when MICs of Nx were compared with that of Cp, sensitivity of the approach was 100% and specificity was 95.95%. The applicability of Nx resistance to screen for decreased susceptibility (low level of resistance) to Ofx and norfloxacin (Nfx) among the *S.* Typhi isolates was also explored in our study and the method emerged useful and significant.

In order to prepare a universally observed guideline for the worldwide surveillance of the emergence and spread of fluoroquinolone-resistant *S.* Typhi isolates, we assessed the applicability of fluoroquinolone disc diffusion test by scattergram analysis between fluoroquinolone MICs and ZDI obtained around 5 *μ*g fluoroquinolone disc. Considering Cp MIC of ≥0.1 *μ*g mL^−1^ as the breakpoint for Cp resistance, Cp ZDI of ≤29 mm resulted in 100% sensitivity and 36.15% specificity in detecting Cp resistance for *S.* Typhi isolates. Similarly, when Ofx MIC of ≥0.5 *μ*g/mL was used as a breakpoint for Ofx resistance, Ofx ZDI of ≤29 mm results in 100% sensitivity and 51.2% specificity in screening for Ofx resistance among the isolates. In case of Nfx, when MIC of ≥0.1 *μ*g/mL was taken as the breakpoint, Nfx ZDI of ≤23 mm produced 100% sensitivity and 49.22% specificity in detecting reduced Nfx susceptibility among *S.* Typhi isolates.

The therapeutic efficacy of ceftriaxone (Ct) against the enteric fever patients infected with Cp-resistant *S*. Typhi had been checked by susceptibility testing with Ct for the isolates; the *in vitro* efficacy of cefotaxime (Cf) was also assessed. We observed a correlation between the results of disc diffusion and agar dilution testing for susceptibility to Ct and Cf by scattergram analysis between MICs and ZDI. For the isolates, the MICs of Ct and Cf were 0.005–0.2 *μ*g mL^−1^ and 0.005–0.1 *μ*g mL^−1^, respectively, and ZDIs obtained around 30 *μ*g Ct and 30 *μ*g Cf discs were 26–41 mm and 24–42 mm, respectively. The isolates showed excellent *in vitro* susceptibility to aminoglycosides (Amikacin; Ak, and gentamicin; G).

The acquisition and spread of multidrug resistance among *S*. Typhi isolates constitute a major threat in modern medicine. Therefore, in order to prepare cost-effective treatment regimen of enteric fever due to the infection of Cp-resistant *S.* Typhi, combination effect of Cp with various other antibiotics has been evaluated in the present study by (a) agar dilution checker board, (b) double disk synergy, and (c) time kill methods. The current findings represent the first time that an antimicrobial, here Cp, has its activity significantly increased with Ax. This view is supported by very low fractional inhibitory concentration (FIC) values of Cp (0.004–0.256 *μ*g mL^−1^) in combination with amoxicillin (Ax). The Cp-trimethoprim (TMP) combination exhibited synergistic effect for all the isolates tested (FIC indices 0.140–0.483). The interaction between Cp and G was found to be synergistic against *S.* Typhi, since, in this study, the calculated FIC indices ranged from 0.121 to 0.216. The Cp-cefazolin (Cz) combination resulted in both synergistic (for isolates with high Cz MICs) and additive (for isolates with low Cz MICs) effects. Synergy was defined as the FIC index ≤0.5, addition as an FIC index 0.5–4, and antagonism as an FIC index >4 [8], and the overall interaction of Cp in combination with other four antibiotics are represented in Table 1.

## 3. Conclusion

In Kolkata (India), enteric fever due to the infection of *S.* Typhi A is endemic. A conjugative plasmid (approximately of 55 kb) was involved mediating resistance to A, C, Co, and T in outbreak causing as well sporadic MDR isolates of *S.* Typhi, while antibiotic-sensitive isolates were plasmidless. Previous report indicated plasmids of molecular weight 25.4 and 62.5 kb to be more frequent among strains with the resistance pattern ACoCT [9]. In *S.* Typhi, the R plasmid is an unstable plasmid that may appear or disappear at any time resulting in the emergence of drug-resistant or drug-sensitive isolates, and its acquisition (from other enteric bacteria like *E. coli*, *P. vulgaris*, *K. pneumoniae*) is mainly due to antibiotic load exposed to them during therapy.

Cp should be the antibiotic of choice for MDR typhoid fever. Longer course of Ofx therapy might be instituted in the treatment of MDR cases showing nonresponsiveness to Cp. Present finding underlines the importance of Ct for treating MDR and Cp-resistant enteric fever cases, and it should be used only if the first- and second-line antibiotics fail to evoke a satisfactory response or if the isolate is resistant to Nx. However, the Ct resistance is anticipated [10]. Emergence of fluoroquinolone-resistant *S.* Typhi isolates showing sensitivity to C, A, and Co provides a strong case for reconsidering the use of the first line of antibiotics for treatment of enteric fever in this region. Another study from Kolkata has indicated similar sensitivities (85.6% for A, 83.6% for C, 83.1% for Co) [11].

Either the MIC breakpoints or the equivalent ZDI suggested by the NCCLS did not detect fluoroquinolone resistance of *S*. Typhi. Nx susceptibility has been validated as a screening test for reduced susceptibility to Cp, and Nx-resistance is associated with a high MIC of Cp, which in turn is associated with treatment failure; earlier authors also reported similar phenomenon [12]. The reference laboratories should assess the applicability of the disc diffusion test with fluoroquinolones in detecting decreased susceptibility to these drugs by scattergram analysis. In enteric fever combined antibiotic therapy may be introduced in order to combat multidrug resistance of *S*. Typhi and also to prevent emergence of resistance to fluoroquinolones and newer antibiotics such as third-generation cephalosporins. Thus, physicians should be aware about the overuse and misuse of fluaroquinolones and cephalosporins in the treatment of typhoid fever to overcome the burden of drug resistance, and with Ct emerging as the sole defense against Nx-resistant *S*. Typhi infection, it should be instituted only in the event of nonresponsiveness to Cp [3]. The data generated from the entire study is thus expected to be helpful in the introduction of new way of evaluation of drug resistance of *S*. Typhi and treatment protocol, benefiting a large number of people of this region of the world, where enteric fever is a major public health problem.

## Figures and Tables

**Table 1:** Interaction of Cp (ciprofloxacin) in combination with G (gentamicin), trimethoprim (TMP), amoxicillin (Ax) and cefazolin (Cz) against *S. enterica* serovar Typhi isolates.

Antibiotic combination	MIC (*μ*g/mL)	FIC (*μ*g/mL)	FIC index
Cp-G	Cp: 0.75–1.25	Cp: 0.008–0.032	0.121–0.216
	GM: 0.75–2	GM: 0.1–0.2	
Cp-TMP	Cp: 0.5–1	Cp: 0.025–0.125	0.14–0.483
	Tm: 10–125	Tm: 5–10	
Cp-Ax	Cp: 1–1.25	Cp: 0.004–0.256	0.504–0.832
	Ax: 0.5–16	Ax: 0.25–10	
Cp-Cz	Cp: 0.5–1.25	Cp: 0.075–0.25	0.35–0.916
	Cz: 2.5–60	Cz: 1.25–2.5	

FIC: fractional inhibitory concentration; MIC: minimum inhibitory concentration.
